# Supplementary material for: Pain distribution can be determined by classical conditioning
Source: Pain. 2025 Mar 18;166(10):2300–9. doi: 10.1097/j.pain.0000000000003586 (PMC12444901; doi:10.1097/j.pain.0000000000003586)
Supplement: SUPPLEMENTARY MATERIAL [file jop-166-2300-s001.pdf]

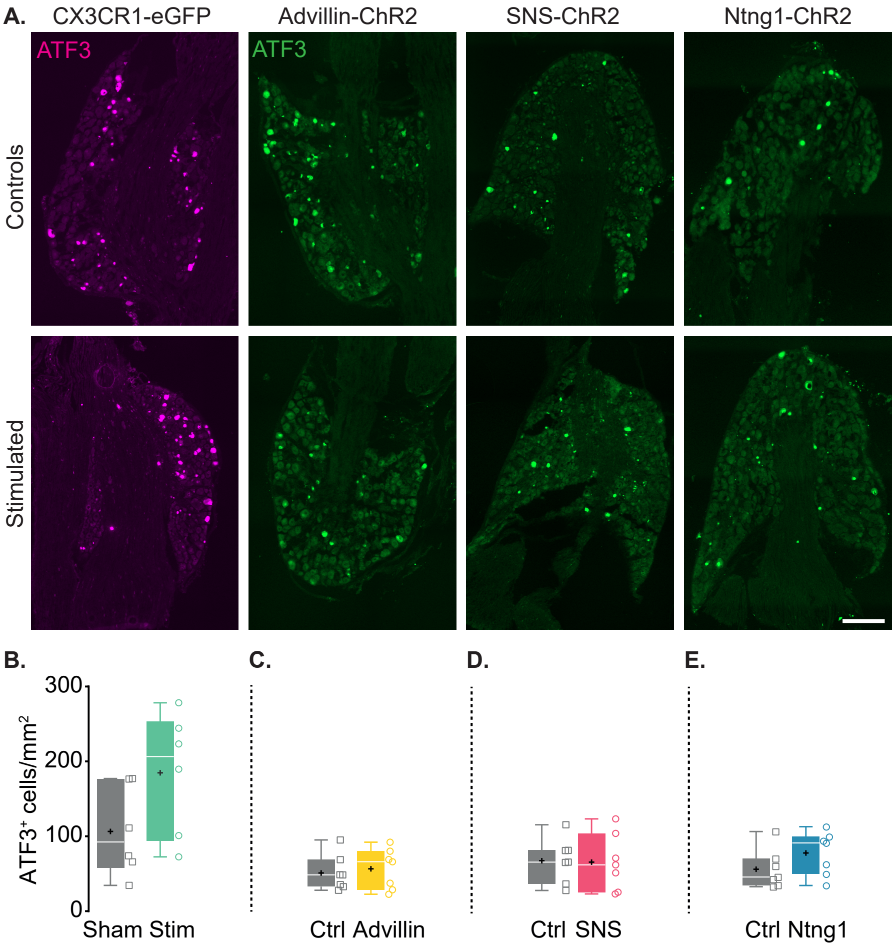

Figure S1. Electrical/optogenetic stimulation of sciatic nerve does not induce neuronal injury.

A. Representative images of ATF3+ cells in DRG from controls or electrically/optogenetically stimulated mice. Scale bar 200 µm

B. Number of ATF3+ cells per mm2 in sham or stim group. *Mean ± SD: Sham = 106.6 ± 59.7, Stim = 185 ± 81.8. Unpaired t test, two tailed, p=0.0872*

C. Number of ATF3+ cells per mm2 in control or Advillin group. *Mean ± SD: Ctrl = 50.5 ± 23.7, Advillin = 56 ± 27. Unpaired t test, two tailed, p=0.6944*

D. Number of ATF3+ cells per mm2 in control or SNS group. *Mean ± SD: Ctrl = 65.8 ± 29.6, SNS = 63.9 ± 37.6. Unpaired t test, two tailed, p=0.9196*

E. Number of ATF3+ cells per mm2 in control or Ntng1 group. *Mean ± SD: Ctrl = 56 ± 26, Ntng1 = 77.7 ± 29.1. Unpaired t test, two tailed, p=0.1675*

Stim, stimulated. Ctrl, control littermates.

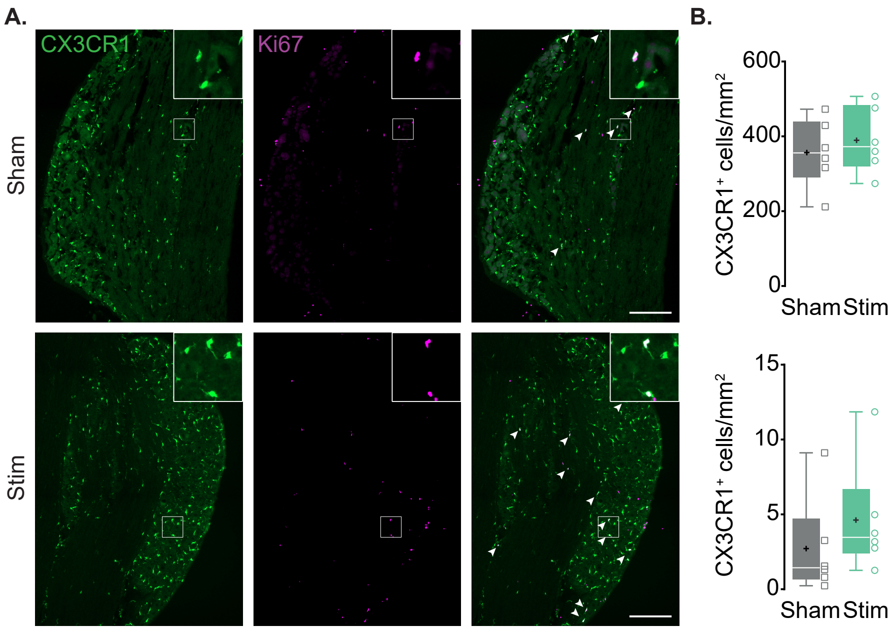

Figure S2. Electrical stimulation of sciatic nerve does not induce macrophages reactivity in the DRGs

A. Representative images of L4 DRG with CX3CR1+ cells (macrophages, in green), Ki67+ (proliferating cells, in purple) and merged (proliferating macrophages, in white). Arrowheads point to colocalization examples. Scale bar 200 µm

B. Top. Number of CX3CR1-eGFP+ cells per mm2 in sham or stim group. *Mean ± SD: Sham = 357 ± 91.4, Stim = 389.4 ± 87.6. Unpaired t test, two tailed, p=0.5444*. Bottom. Number of CX3CR1-eGFP+Ki67+ cells per mm2 in sham or stim group. *Mean ± SD: Sham = 2.7 ± 3.3, Stim = 4.6 ± 3.7. Unpaired t test, two tailed, p=0.369*

Stim, stimulated.

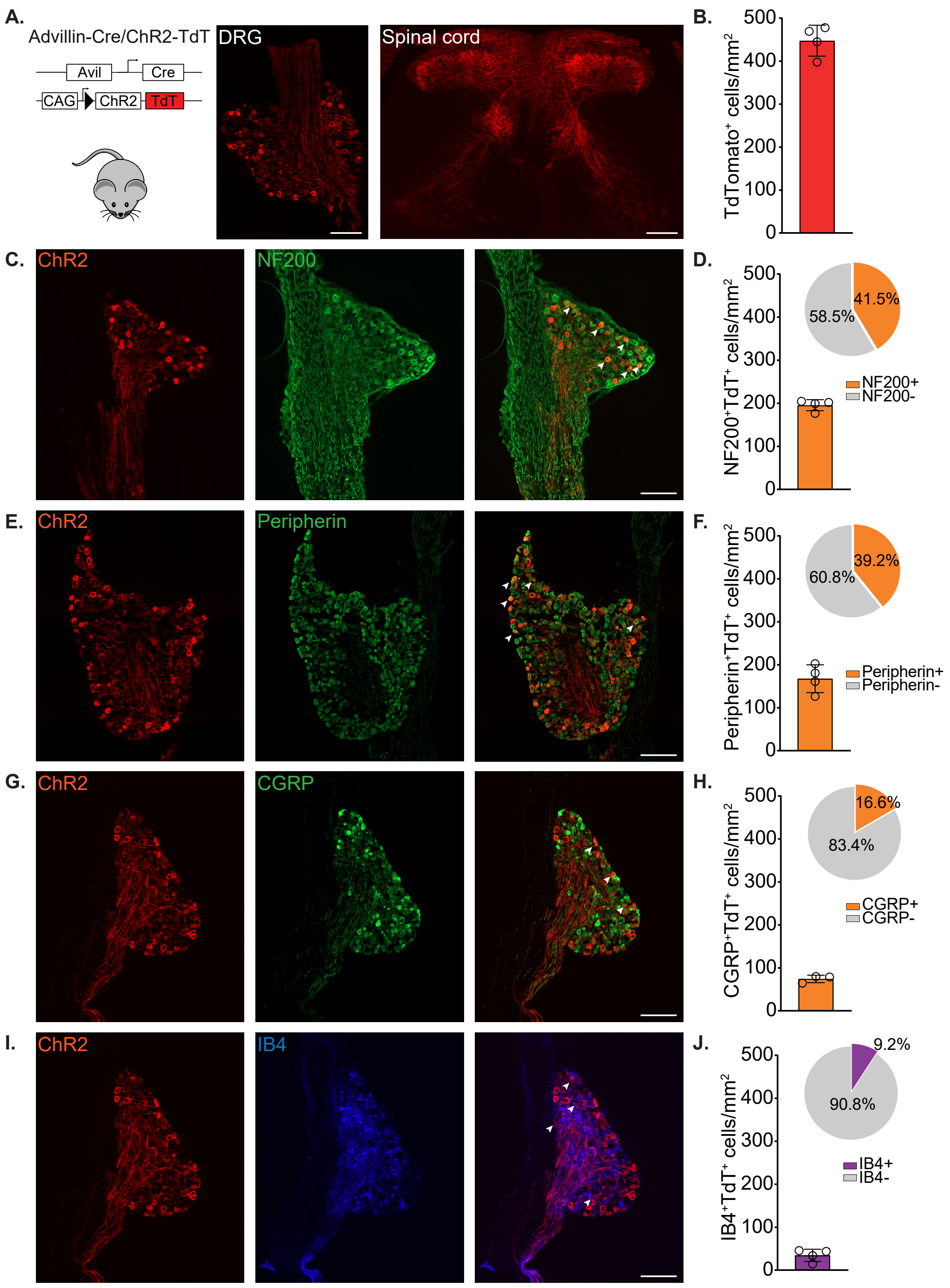

A.
Advillin-Cre/ChR2-TdT
Avil
Cre
CAG
ChR2
TdT
DRG
Spinal cord
B.
TdTomato+ cells/mm2
C.
ChR2
NF200
D.
NF200+TdT+ cells/mm2
41.5%
58.5%
NF200+
NF200-
E.
ChR2
Peripherin
F.
Peripherin+TdT+ cells/mm2
39.2%
60.8%
Peripherin+
Peripherin-
G.
ChR2
CGRP
H.
CGRP+TdT+ cells/mm2
16.6%
83.4%
CGRP+
CGRP-
I.
ChR2
IB4
J.
IB4+TdT+ cells/mm2
9.2%
90.8%
IB4+
IB4-

Figure S3. Anatomical characterization of Advillin-ChR2 mouse line

A. Construction of the Advillin-ChR2 transgenic line and representative images showing ChR2-TdTomato expression in DRG and spinal cord.

B. Number of TdTomato+ cells per mm2 in L3-L5 DRGs. *Mean ± SD: 447.6 ± 35.9*

C. Representative images of TdTomato+ cells, NF200+ cells and their colocalization.

D. Number of TdTomato+NF200+ cells per mm2 (*mean ± SD: 195.5 ± 12.8*) and proportion of NF200+ and NF200- cells in ChR2-TdTomato+ population.

E. Representative images of TdTomato+ cells, Peripherin+ cells and their colocalization.

F. Number of TdTomato+Peripherin+ cells per mm2 (*mean ± SD: 167.7 ± 32.4*) and proportion of Peripherin+ and Peripherin- cells in ChR2-TdTomato+ population.

G. Representative images of TdTomato+ cells, CGRP+ cells and their colocalization.

H. Number of TdTomato+CGRP+ cells per mm2 (*mean ± SD: 74.4 ± 8.6*) and proportion of CGRP+ and CGRP- cells in ChR2-TdTomato+ population.

I. Representative images of TdTomato+ cells, IB4+ cells and their colocalization.

J. Number of TdTomato+IB4+ cells per mm2 (*mean ± SD: 34.4 ± 14.2*) and proportion of IB4+ and IB4- cells in ChR2-TdTomato+ population.

Arrowheads point to colocalization examples. Scale bar 200 µm

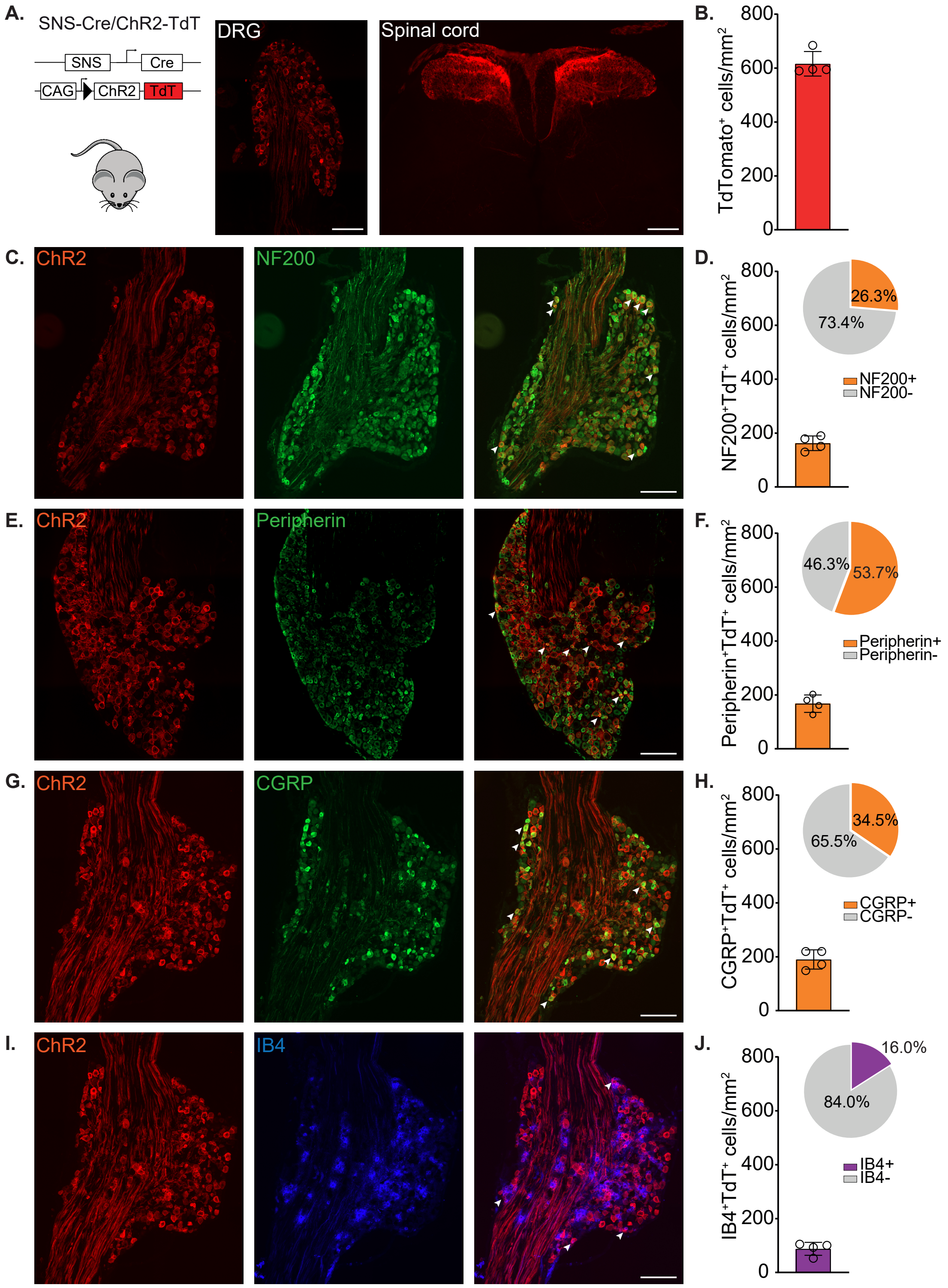
A.
SNS-Cre/ChR2-TdT
SNS
Cre
CAG
ChR2
TdT
DRG
Spinal cord
B.
TdTomato+ cells/mm2
800
600
400
200
0
C.
ChR2
NF200
D.
NF200+TdT+ cells/mm2
26.3%
73.4%
NF200+
NF200-
E.
ChR2
Peripherin
F.
Peripherin+TdT+ cells/mm2
46.3%
53.7%
Peripherin+
Peripherin-
G.
ChR2
CGRP
H.
CGRP+TdT+ cells/mm2
34.5%
65.5%
CGRP+
CGRP-
I.
ChR2
IB4
J.
IB4+TdT+ cells/mm2
16.0%
84.0%
IB4+
IB4-

Figure S4. Anatomical characterization of SNS-ChR2 mouse line

A. Construction of the SNS-ChR2 transgenic line and representative images showing ChR2-Td-Tomato expression in DRG and spinal cord.

B. Number of TdTomato+ cells per mm2 in L3-L5 DRGs. *Mean ± SD: 612.2 ± 45.7*

C. Representative images of TdTomato+ cells, NF200+ cells and their colocalization.

D. Number of TdTomato+NF200+ cells per mm2 (*mean ± SD: 162.4 ± 27.2*) and proportion of NF200+ and NF200- cells in ChR2-TdTomato+ population.

E. Representative images of TdTomato+ cells, Peripherin+ cells and their colocalization.

F. Number of TdTomato+Peripherin+ cells per mm2 (*mean ± SD: 387.8 ± 93*) and proportion of Peripherin+ and Peripherin- cells in ChR2-TdTomato+ population.

G. Representative images of TdTomato+ cells, CGRP+ cells and their colocalization.

H. Number of TdTomato+CGRP+ cells per mm2 (*mean ± SD: 190 ± 35.6*) and proportion of CGRP+ and CGRP- cells in ChR2-TdTomato+ population.

I. Representative images of TdTomato+ cells, IB4+ cells and their colocalization.

J. Number of TdTomato+IB4+ cells per mm2 (*mean ± SD: 87.9 ± 24.2*) and proportion of IB4+ and IB4- cells in ChR2-TdTomato+ population.

Arrowheads point to colocalization examples. Scale bar 200 µm

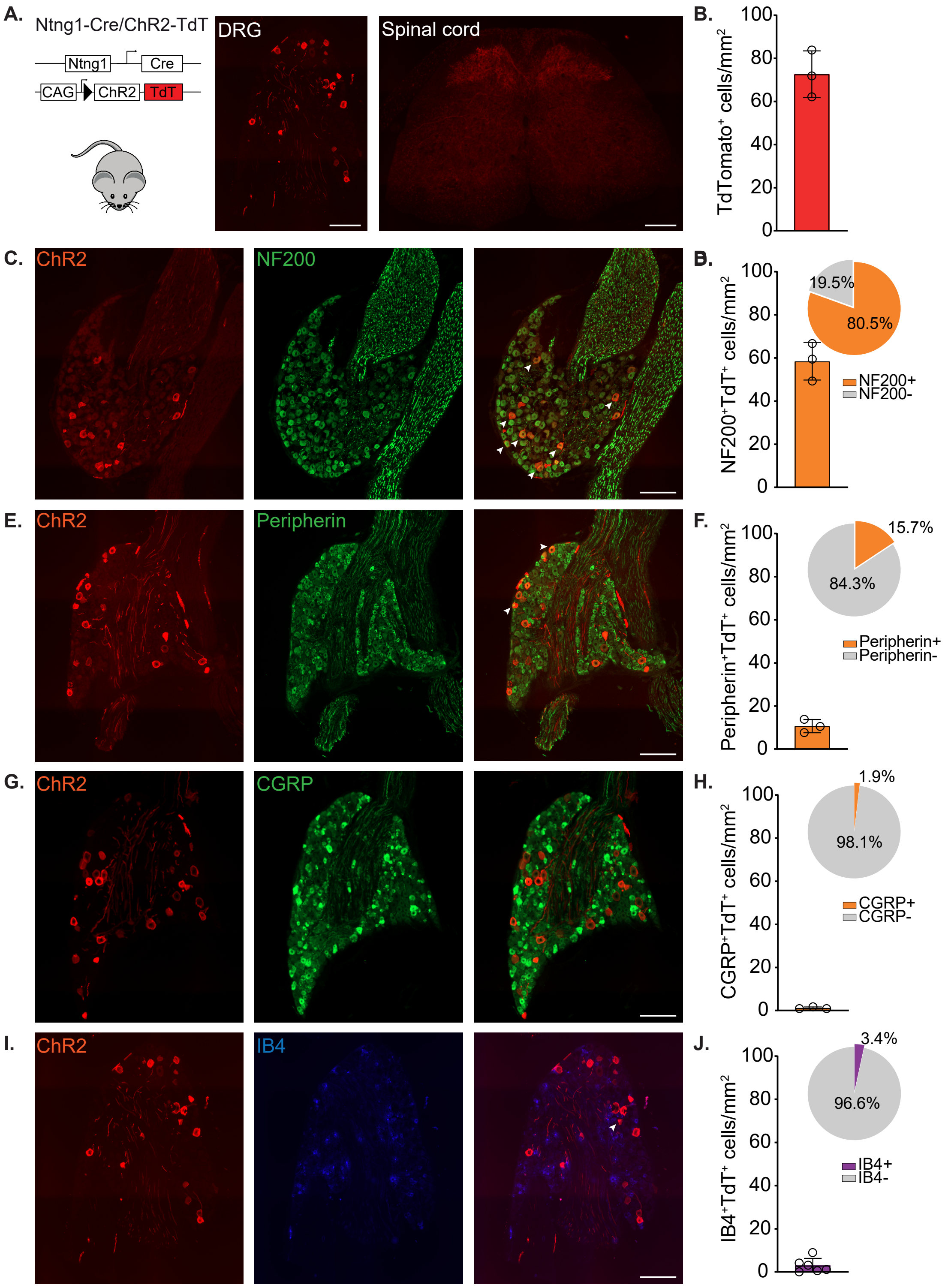
A.
Ntng1-Cre/ChR2-TdT
Ntng1
Cre
CAG
ChR2
TdT
DRG
Spinal cord
B.
TdTomato+ cells/mm2
C.
ChR2
NF200
B.
NF200+TdT+ cells/mm2
19.5%
80.5%
NF200+
NF200-
E.
ChR2
Peripherin
F.
Peripherin+TdT+ cells/mm2
15.7%
84.3%
Peripherin+
Peripherin-
G.
ChR2
CGRP
H.
CGRP+TdT+ cells/mm2
1.9%
98.1%
CGRP+
CGRP-
I.
ChR2
IB4
J.
IB4+TdT+ cells/mm2
3.4%
96.6%
IB4+
IB4-
0
20
40
60
80
100

Figure S5. Anatomical characterization of Ntng1-ChR2 mouse line

A. Construction of the Ntng1-ChR2 transgenic line and representative images showing ChR2-TdTomato expression in DRG and spinal cord.

B. Number of TdTomato+ cells per mm2 in L3-L5 DRGs. *Mean ± SD: 72.6 ± 10.9*

C. Representative images of TdTomato+ cells, NF200+ cells and their colocalization.

D. Number of TdTomato+NF200+ cells per mm2 (*mean ± SD: 58.5 ± 8.7*) and proportion of NF200+ and NF200- cells in ChR2-TdTomato+ population.

E. Representative images of TdTomato+ cells, Peripherin+ cells and their colocalization.

F. Number of TdTomato+Peripherin+ cells per mm2 (*mean ± SD: 10.7 ± 3.1*) and proportion of Peripherin+ and Peripherin- cells in ChR2-TdTomato+ population.

G. Representative images of TdTomato+ cells, CGRP+ cells and their colocalization.

H. Number of TdTomato+CGRP+ cells per mm2 (*mean ± SD: 1.2 ± 0.5*) and proportion of CGRP+ and CGRP- cells in ChR2-TdTomato+ population.

I. Representative images of TdTomato+ cells, IB4+ cells and their colocalization.

J. Number of TdTomato+IB4+ cells per mm2 (*mean ± SD: 3 ± 3.3*) and proportion of IB4+ and IB4- cells in ChR2-TdTomato+ population.

Arrowheads point to colocalization examples. Scale bar 200 µm

Key resources table

REAGENT or RESOURCE	SOURCE	IDENTIFIER
Antibodies		
Rabbit anti-ATF3	Abcam, Waltham, MA, USA	Cat# ab207434, RRID:AB_2734728
Rabbit anti-Ki67	Abcam, Waltham, MA, USA	Cat# ab15580, RRID:AB_443209
Goat anti-Iba1	Abcam, Waltham, MA, USA	Cat# ab5076, RRID:AB_2224402
Mouse anti-NF200	Sigma-Aldrich, Saint-Louis, MO, USA	Cat# N0142 RRID:AB_477257
Rabbit anti-NF200	Sigma-Aldrich, Saint-Louis, MO, USA	Cat# N4142 RRID:AB_477272
Rabbit anti-peripherin	Sigma-Aldrich Inc., Saint-Louis, MO, USA	Cat# AB1530, RRID:AB_90725
Rabbit anti-CGRP	Peninsula Laboratories International, Inc, San Carlos, CA, USA	Cat# T-4032, RRID:AB_518147
Biotinylated anti-IB4	Vector Laboratories, Newark, CA, USA	Cat# B-1205, RRID:AB_2314661
Alexa Fluor 488-conjugated donkey antigoat	Molecular Probes, Eugene, OR, USA	Cat# A11055, RRID:AB_2534102
Cy3-conjugated donkey antigoat	Jackson ImmunoResearch, Ely, UK	Cat# 705-165-147, RRID:AB_2307351
Cy5-conjugated goat anti-rabbit	Invitrogen., Waltham, MA, USA	Cat# A10523, RRID:AB_2534032
Alexa Fluor 647 conjugated donkey antirabbit	Invitrogen., Waltham, MA, USA	Cat# A31573, RRID:AB_2536183
Alexa Fluor 488-conjugated donkey antirabbit	Molecular Probes, Eugene, OR, USA	Cat# A-21206, RRID:AB_2535792
Alexa Fluor 488-conjugated goat antimouse	Molecular Probes, Eugene, OR, USA	Cat# A-11029, RRID:AB_2534088
Alexa Fluor 350-conjugated goat antirabbit	Molecular Probes, Eugene, OR, USA	Cat# A-21068, RRID:AB_141378
AMCA-conjugated Streptavidin	Jackson ImmunoResearch, Ely, UK	Cat# 016-150-084
DAPI	Invitrogen, Waltham, MA, USA	Cat# D-3571, RRID: AB_2307445
Mice line		
Mouse: C57BL/6J	Charles River, Wilmington, MA, USA	strain code 680, C57Bl6j
Mouse: B6.129P-Cx3cr1tm1Litt/J	Jackson Laboratory, Bar Harbor, ME, USA	JAX #005582 RRID:IMSR_JAX:005582
Mouse: C57BL/6-Tg(Scn10a::Cre)1Rkun	Agarwal, N., Offermanns, S., & Kuner, R. (2004)	N/A
Mouse: B6;D2-Tg(Avil-cre)1Phep/Cnrm	EMMA repository	#05542 RRID:IMSR_EM:05542

Mouse: Ntng1-Cre	Bolding, K. A., Nagappan, S., Han, B. X., Wang, F., & Franks, K. M. (2020).	N/A
Mouse: B6.Cg-Gt(ROSA)26Sortm27.1(CAG-COP4*H134R/tdTomato)Hze/J	Jackson Laboratory, Bar Harbor, ME, USA	JAX #012567 RRID:IMSR_JAX:012567
Software and algorithms		
MATLAB R2020b	The MathWorks, Natick, MA, USA	RRID:SCR_001622
Zen3.3 (blue edition)	Carl Zeiss Microscopy GmbH, Jena, Germany	RRID:SCR_013672
GraphPad Prism 9	GraphPad Software Inc., San Diego, CA, USA	GraphPad Prism, RRID:SCR_002798
Fiji (ImageJ Software) v1.53c	Schindelin et al., 2012	Fiji, RRID:SCR_002285
CellSens Software v3.2	Olympus, Tokyo, Japan	RRID:SCR_014551
pClamp 10.3	Molecular Devices, San Jose, CA, USA	RRID:SCR_011323
Reagents		
Paraformaldehyde	Sigma-Aldrich Inc., Saint-Louis, MO, USA	P6148
Sucrose (D+ saccharose)	AppliChem, Darmstadt, Germany	#A2211
Saline 0.9% solution	Bichsel AG, Interlaken, BE, Switzerland	FE1001339
Tissue-Tek O.C.T. Compound	Sakura Finetek, Alphen aan den Rijn, Netherlands	#4583
Glycerol	Merck, Darmstadt, Germany	#356352
Ethylene Glycol	Supelco,	1.09621.1000
Phosphate buffered saline	Thermo Fisher Scientific Inc., Waltham, MA, USA	#10010-015
Phosphate buffered saline 10X	Bichsel AG, Interlaken, BE, Switzerland	100 0 325 00
Mowiol 4–88 medium	Merck Millipore, Burlington, MA, USA	#475904
Triton-X-100	Sigma-Aldrich Inc., Saint-Louis, MO, USA	#T9284
Normal goat serum	Vector Laboratories, Newark, CA, USA	S-1000
Normal horse serum	Vector Laboratories, Newark, CA, USA	S-2000
DMEM	Gibco, Billings, MT, USA	41965-039
Heat inactivated fetal bovine serum	Gibco, Billings, MT, USA	10082
Penicillin-streptomycin	Sigma-Aldrich Inc., Saint-Louis, MO, USA	P0781
NaCl	Sigma-Aldrich Inc., Saint-Louis, MO, USA	#S9625

KCl	Sigma-Aldrich Inc., Saint-Louis, MO, USA	#P9333
$CaCl_2$*$2H_2O_2$	Merck Millipore, Burlington, MA, USA	#102382
$MgCl_2$*$6H_2O_2$	Merck Millipore, Burlington, MA, USA	#105832
HEPES	AppliChem, Darmstadt, Germany	#A1069
Glucose D-(+)	Sigma-Aldrich Inc., Saint-Louis, MO, USA	#G7021
ChoCl	Sigma-Aldrich Inc., Saint-Louis, MO, USA	#C1879
NaOH	Sigma-Aldrich Inc., Saint-Louis, MO, USA	#S8045
EGTA (Ethylene Glycol Tetra Acetic Acid)	Sigma-Aldrich Inc., Saint-Louis, MO, USA	#E4378
KOH	Sigma-Aldrich Inc., Saint-Louis, MO, USA	P5958-250G
Drugs		
Minocycline hydrochloride	Sigma-Aldrich Chemie GmbH	Cat #M9511
Pentobarbital	Streuli Pharma, USA	V102013
Isoflurane	Piramal, Mumbai, Maharashtra, India	G45C19A
Material/Equipment		
DS3 Current Stimulator	Digitimer Ltd, Hertfordshire, England	NA
Grass SD9 B Square Pulse Stimulator	Grass Instruments Company, West Warwick, RI, USA	NA
Dual fiber-optic patch cord	Doric Lenses Inc, Québec, Canada	D204-2030-2 Code 200/220/900-0.37
LRD-0470 Collimated Diode Laser System	Laserglow Technologies	D4B2003FX
Von Frey filaments	Bioseb, Vitrolles, France	BIO-VF-M
Hargreaves apparatus	IITC Life Science Inc., Woodland Hills, CA, USA	Cat #390G
Zeiss Axioscan Z.1 slide scanner	Zeiss, Oberkochen, Germany	RRID:SCR_020927
Leica Stellaris 8	Leica Microsystems, Wetzlar, Germany	RRID:SCR_024664
Fire-polished borosilicate glass pipettes	Sutter Instrument CO, Novato, CA, USA	Cat# BF150-86-7.5HP
Flaming/brown micropipette puller P-97	Sutter Instrument CO, Novato, CA, USA	Model P-97
MF200-2 microforge	World Precision Instruments, Sarasota, FL, USA	MF200-2
H4 platinum/iridium wire	World Precision Instruments, Sarasota, FL, USA	MF-200 H4

W30S-LED Revelation III	LW Scientific, Lawrenceville, GA, USA	W30S
Multiclamp Axon Amplifier 700B	Molecular Devices, San Jose, CA, USA	#700B
Digidata 1440A	Molecular Devices, San Jose, CA, USA	#1440A
BX51W1 Microscope	Olympus, Tokyo, Japan	BX51
ORCAFlash 2.8 digital camera	Hamamatsu Photonics, Shizuoka, Japan	#820504
CoolLED pE-340fura	CoolLED Ltd, Andover, UK	#pE-340 fura
LED eGFP pE-300 filterset	CoolLED Ltd, Andover, UK	#E3990113, Exciter: 460/30; Emitter: 520/40
SevenCompact S210	Mettler Toledo, Columbus, OH, USA	S210
Osmometer 3320	Advanced Instruments Inc, Norwood, MA, USA	3320
Epredia™ SuperFrost Plus™ Adhesion slides	Epredia, Breda, Netherlands	J1800AMNZ
Microscope cover glasses	VWR International, Radnor, PA, USA	631-1577
